# Testicular Torsion in Monorchism Diagnosed with Point-of-care Ultrasound: A Case Report

**DOI:** 10.5811/cpcem.2020.12.48944

**Published:** 2021-01-22

**Authors:** Chad Correa, So Onishi, Eric Abrams

**Affiliations:** Kaiser Permanente San Diego, Department of Emergency Medicine, San Diego, California

**Keywords:** Testicular torsion, point-of-care ultrasound, testicular ultrasound

## Abstract

**Introduction:**

Emergency department physicians should incorporate point-of-care-ultrasound (POCUS) in the assessment of patients presenting with acute scrotal pain for rapid identification of the time sensitive urologic emergency, testicular torsion.

**Case Report:**

A 20-year-old otherwise healthy male, with a history of monorchism, presented to the emergency department with vague testicular pain. A POCUS was performed, which demonstrated attenuated arterial flow of the patient’s single testicle as well as twisting (“whirlpool sign”) of the spermatic cord, both highly specific ultrasonographic findings of testicular torsion.

**Conclusion:**

These findings expedited definitive management resulting in the salvage of the single ischemic testicle.

## INTRODUCTION

Scrotal and testicular complaints comprise at least 0.5% of all emergency department (ED) visits. Of the various etiologies of testicular discomfort, only testicular torsion is seen as a true, time-sensitive urologic emergency.^1^ Testicular torsion occurs when a testicle twists around the spermatic cord, resulting in compromised blood flow to the testicle and resultant downstream tissue ischemia.^1^ The extent of testicular rotation has also been directly correlated with the time to testicular necrosis and, therefore, the probability of salvage.^2^ The incidence of testicular torsion is 3.8 in 100,000 males up to the age of 18. It often occurs during sleep and in the absence of trauma.^3^

The congenital absence of one testes, monorchism, is considered rare.^4^ To have monorchism and torsion of the single viable testicle is not only extremely uncommon, but exceedingly more urgent as necrosis would result in long-term sequelae including infertility. With the recent incorporation of point-of-care ultrasound (POCUS) as a core competency in the emergency medicine training curriculum,^5^ POCUS has now become a standard tool in the emergency physician’s repertoire. Additionally, since ultrasound is the ideal imaging modality to evaluate the scrotum and its contents,^6^ emergency physicians should include POCUS in the workup for the time-sensitive diagnosis of testicular torsion, especially in resource-poor settings.

## CASE REPORT

A 20-year-old male with a past medical history of monorchism presented to the ED with one hour of sudden onset, cramping-like, progressively worsening, 10 out of 10 testicular pain that woke him from sleep. The pain had been present for approximately four hours. He denied any trauma, dysuria, or concerning sexual history. When questioned on the medical history of his single testis, he reported having only one testicle from birth, without understanding the etiology or ever having been evaluated for it.

**Image 1:**
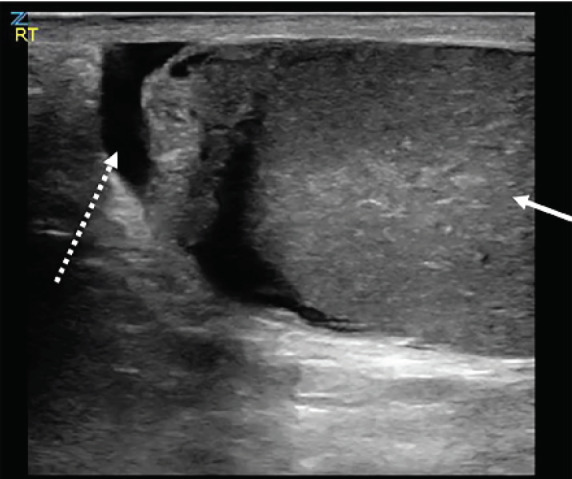
Point-of-care ultrasound demonstrating heterogenous testicle (solid arrow) with simple hydrocele (dashed arrow).

**Image 2:**
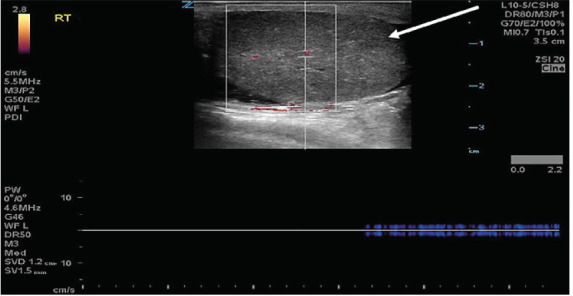
Point-of-care ultrasound demonstrating heterogenous testicle (arrow) with limited, attenuated arterial flow on spectral Doppler.

His vital signs upon presentation demonstrated a heart rate of 79 beats per minute, systolic blood pressure of 145 millimeters mercury, respirations of 18 breaths per minute, oxygen saturation of 99% on room air, and an oral temperature of 97.7° Fahrenheit. Physical exam demonstrated a single, firm, edematous testicle with a horizontal lie that was significantly tender to palpation. There was also a tender, firm spermatic cord. There was no surrounding scrotal edema. His abdomen was soft and nontender, and there was no evidence of inguinal hernia. A point-of-care ultrasound was performed, which demonstrated a heterogenous, single right testicle located within a surrounding simple hydrocele (Image 1). The testicle had limited, non-pulsatile flow on spectral Doppler ultrasound (Image 2).

**Image 3:**
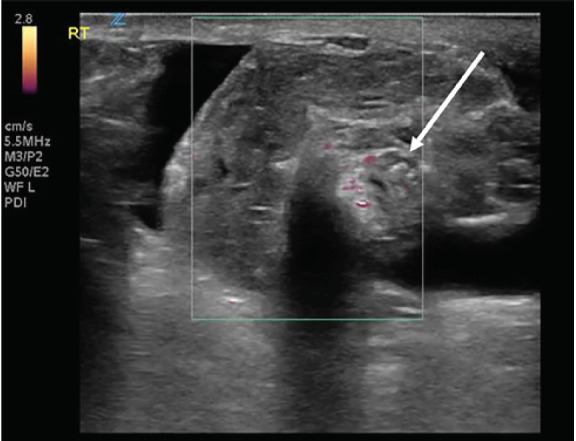
Point-of-care ultrasound of the spermatic cord demonstrating “whirlpool sign” (arrow) and diminished vascular flow on power Doppler ultrasound.

The epididymis appeared unremarkable. The spermatic cord was identified, and when tracked proximally had an abrupt change in its course with a spiral twist in the scrotal sac, referred to as the “whirlpool sign” (Image 3).

Unfortunately, since the patient had a history of monorchism there was no contralateral testicle for comparison of vascular flow or echotexture. Given the patient’s complaint, exam, and sonographic findings, a diagnosis of testicular torsion was made. Urology was emergently consulted, and the patient was scheduled for immediate surgery. A formal comprehensive ultrasound was performed pending the consultant arrival, which reiterated findings described in the POCUS. The patient was taken to the operating room and had a successful detorsion and orchidopexy, and was ultimately discharged home. Outpatient follow-up evaluation performed by urology five weeks after surgery demonstrated a preserved, single viable testicle.

## DISCUSSION

Ultrasound is the imaging modality of choice for the diagnosis of testicular torsion. A definitive sign, as observed in this case, is twisting of the spermatic cord, also called the “whirlpool sign.”^7^ The whirlpool sign is a reliable and direct sonographic finding that implies torsion of the spermatic cord and testis.^8^ Other ultrasonographic findings include absent or attenuated arterial blood flow, testicular edema, heterogeneous echotexture, reactive hydrocele, and reactive thickening of scrotal skin.^9^–^10^ If testicular torsion is suspected and bedside manual detorsion is performed to restore flow to the affected testicle, repeat ultrasound imaging may demonstrate hyperemic flow to the affected testicle.^11^

**CPC-EM Capsule**

What do we already know about this clinical entity?

Testicular torsion is a time sensitive emergency.

What makes this presentation of disease reportable?

The use of point-of-care ultrasound to expedite definitive management of torsion in a patient with one viable testicle to preserve fertility.

What is the major learning point?

Point-of-care ultrasound can help confirm testicular torsion rapidly at the bedside.

How might this improve emergency medicine practice?

Point-of-care ultrasound should be utilized in patients who present with acute testicular pain to facilitate earlier diagnosis, surgical consultation, and definitive management.

When performing a scrotal ultrasound, the testicle in question can be compared to the contralateral testicle. Differences in echotexture, testicular size, and vascular flow can aid in the diagnosis of testicular torsion. What makes this case unusual is the patient’s history of monorchism; thus, the ultrasonographic findings could not be compared to a healthy testicle. The appearance of the twisted spermatic cord and absence of testicular flow as visualized by POCUS made the diagnosis of testicular torsion extremely likely even without the ability to compare our findings to a contralateral testicle.

## CONCLUSION

Point-of-care ultrasound helps emergency physicians improve the accuracy of their diagnoses and provides better overall patient care. If a patient presents with testicular or scrotal pain, using POCUS to examine for attenuated or absent vascular flow with the “whirlpool sign” of the spermatic cord may aid in confirming the diagnosis of testicular torsion, thus preventing delay in care and providing consultants with more objective data, especially in resource-poor settings where formal imaging may not be readily available.
